# Effect of Pilates Training on Alpha Rhythm

**DOI:** 10.1155/2013/295986

**Published:** 2013-06-19

**Authors:** Zhijie Bian, Hongmin Sun, Chengbiao Lu, Li Yao, Shengyong Chen, Xiaoli Li

**Affiliations:** ^1^Institute of Electrical Engineering, Yanshan University, Qinhuangdao 066004, China; ^2^College of Physical Education, Yanshan University, Qinhuangdao 066004, China; ^3^National Lab of Cognitive Neuroscience and Learning, Beijing Normal University, Xin Jie Kou Wai Avenue, Haidian District, Beijing 100875, China; ^4^College of Computer Science and Technology, Zhejiang University of Technology, Hangzhou 310023, China

## Abstract

In this study, the effect of Pilates training on the brain function was investigated through five case studies. Alpha rhythm changes during the Pilates training over the different regions and the whole brain were mainly analyzed, including power spectral density and global synchronization index (GSI). It was found that the neural network of the brain was more active, and the synchronization strength reduced in the frontal and temporal regions due to the Pilates training. These results supported that the Pilates training is very beneficial for improving brain function or intelligence. These findings maybe give us some line evidence to suggest that the Pilates training is very helpful for the intervention of brain degenerative diseases and cogitative dysfunction rehabilitation.

## 1. Introduction

Pilates was created in the 1920s by physical trainer Joseph H. Pilates and has been developed based on the Eastern and Western health preservation methods, such as Yoga and Taichi. This exercise is suitable for all the people and may be one of the most attractive fitness trainings [1, 2]. Pilates exercise was found to be able to correct body posture, relax the waist and neck, solve the problem of shoulder, and reduce fat of arm and abdomen [3–5]. Pilates can improve the blood circulation and cardiopulmonary function as the exercise is dominated by the rhythmic breath, particularly the lateral thoracic breathing that can effectively promote the exchange of oxygen. The Pilates has been proven to impact personal autonomy [6], pain control [1], improved muscle strength [7], flexibility [8], and motor skills [9]. Physical activity can be considered as an approach to improve organic conditions and prevent physical degeneration [10]. Further studies suggest that Pilates can release the stress of mind, increase brain's oxygen supply, and enhance brain function [11, 12], and studies in aged samples also suggest that Pilates is beneficial to mental state, including sleep quality, emotion, and self-confidence [2].

However, the direct evidence of Pilates on brain activity such as electroencephalographic (EEG) is lacking. In this study, we recorded resting-state EEG signals before and after Pilates exercise. We concentrated on the analysis of alpha rhythm (8–13 Hz) changes of the EEG, which is associated with the intelligence. The aim is to demonstrate whether or not Pilates can impact the brain functions or intelligence.

## 2. Methods 

### 2.1. Subjects

After providing informed consent, five healthy postgraduate girls (mean age 24 ± 1 years) voluntarily participated in this study. They were free to withdraw from the experiments at any time. All subjects included in this experiment were right-handed, nonathletes, and had never been suffering from neurological and psychiatric disorders. The study was approved by the local ethics committee, and all participants gave written informed consent for this study.

### 2.2. Pilates Training

The five girls were trained with Pilates four sessions a week (Monday, Tuesday, Thursday, and Friday) in a well-ventilated room, at least 90 minutes per session. For the first three weeks, they were taught Pilates movements step by step, and they reviewed the former movements in each training session and were corrected by the coach after learning the new ones. After they were taught a total of 24 movements, they practiced for 4–6 times in each session, and they were instructed to perform the sequences as accurately and smoothly coupled with breathing. The training lasted for 10 weeks. And the resting-state EEG rhythms were recorded with eyes closed before Pilates training and after each two weeks training. 

### 2.3. Data Acquisition

EEG recordings were performed at six different time points. The first recording was performed just prior to the onset of training week (week 0). After each two weeks training, there was one recording, such as week 2, week 4, week 6, week 8, and week 10. During recordings, the subjects were asked to close their eyes and sit in a comfortable armchair, who were relaxed and awake in a dim room for 5 minutes during each recording. 

The EEG data acquisition was performed with Neuroscan EEG/ERP recording system amplifiers (SynAmps2) with 64 Ag/AgCl surface electrodes, which were fixed in a cap at the standard positions according to the extended international 10–20 system, and with 32 bit SCAN4.5 acquisition system that could also be used to continuously view the EEG recordings. A reference electrode was placed between Cz and CPz, and ground electrode was placed between FPz and Fz. Horizontal and vertical electrooculograms (EOG) were recorded as well. The EEG was recorded with unipolar montages except for the EOG with bipolar montages. The impedances of all electrodes were <10 kΩ. During the recording, the data was band-pass filtered in the frequency range 0.05–200 Hz and sampled at 2 KHz. Digital conversion of the measured analog signals was accomplished with a 24 bit digitizer. 

### 2.4. Data Analysis

**Figure 1:**
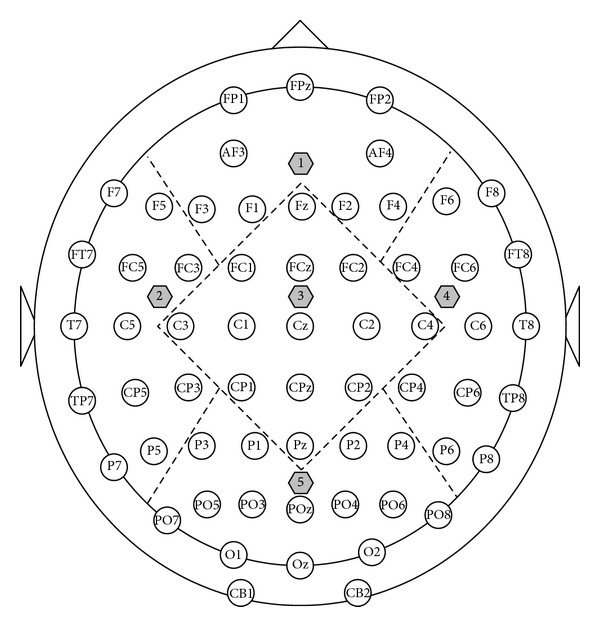
Extended 10–20 electrodes system and area electrodes' partition. The dotted lines divided the whole into 5 regions: the numbers 1, 2, 3, 4, and 5 separately denote the frontal, left temporal, central, right temporal, and posterior regions, respectively.

In this study, the alpha rhythm (8–13 Hz) in the EEG recordings was concentrated on. In order to detect the alpha rhythm's changes over different regions, the brain was divided into five regions: frontal, left temporal, central, right temporal, and posterior (see Figure 1). Power spectral density and global synchronization index (GSI) at the alpha frequency band were computed in all regions. 

#### 2.4.1. Preprocessing for EEG

The raw EEG data was analyzed offline using EEGLAB (http://sccn.ucsd.edu/eeglab/ [13]). It was rereferenced to M1 (left mastoid process) and M2 (right mastoid process), the two EOG channels were extracted, the band-pass filter (8–13 Hz) was initially used to include the frequency band of interest, and then the data was resampled to 250 Hz for further analysis. 

#### 2.4.2. Spectral Analysis

After preprocessing, we chose EEG data of 4 minutes for analysis. Power spectral density (PSD) was estimated using pwelch method, which has a better noise performance compared with other power spectra estimation methods. The PSD was calculated using 10s epochs for each signal. Each epoch was divided into overlapping segments using periodic 10-s hamming window with 50% overlap. And then the peak power and peak power frequency were calculated for the alpha band in each epoch. Outliers rejection was performed using generalized extreme studentized deviate (GESD) [14] for all epochs in each channel. The remained epochs were averaged. 

The PSD for each channel in all frequency bands was obtained. In order to estimate the changes of peak power and corresponding frequency during the Pilates training over different regions and the whole brain, the PSD was averaged over each region and the whole brain. 

#### 2.4.3. GSI

Synchronization is known as a key feature to evaluate the information process in the brain. For long EEG data, global synchronization index (GSI) can reveal the true synchronization features of multivariable EEG sequences better than other methods [15].

To eliminate the effect of amplitude, the EEG signals preprocessed need to be normalized by

(1) $$\begin{array}{c} Z=\left\{z_{i}\left(n\right)\right\}\;\left(i=1,\ldots ,M;n=1,\ldots ,T\right),\\ x_{i}\left(n\right)=\frac{\left(z_{i}\left(n\right)-\langle Z_{i}\rangle \right)}{\sigma_{i}},\\ X=\left\{x_{i}\left(n\right)\right\}, \end{array}$$

where *Z* is considered as the multivariate EEG data, *M* is the number of channels, *n* is the number of data points in time window *T*, *x*_*i*_(*n*) is the normalized signal, and *X* is a vector of *x*_*i*_(*n*), and 〈*Z*_*i*_〉 and *σ*_*i*_ are the mean and standard deviation of *z*_*i*_(*n*), respectively.

To calculate the GSI of multivariate EEG data, a phase correlation matrix **C** was constructed. The phase of the each EEG series is estimated using continuous wavelet transform. The phase difference of two EEG traces is defined by

(2) $$\begin{array}{c} \Delta \varphi_{x_{i}x_{k}}^{w}\left(s,\tau \right)=\varphi_{x_{i}}^{w}\left(s,\tau \right)-\varphi_{x_{k}}^{w}\left(s,\tau \right)\;\left(k=1,\ldots ,M\right). \end{array}$$

Then, the phase synchronization is calculated by

(3) $$\begin{array}{c} \gamma_{ik}=\left|{\langle e^{j\Delta \varphi_{x_{i}x_{k}}^{w}(s,\tau )}\rangle }_{T}\right|\in \left[0,1\right], \end{array}$$

where 〈·〉_*T*_ indicates the average of the time window *T*. *γ*_*ik*_ indicates the phase synchronization of signals *x*_*i*_(*n*) and *x*_*k*_(*n*). For all EEG series, a phase correlation matrix can be written as **C** = {*γ*_*ik*_}.

Then, the eigenvalue decomposition of **C** is defined as follows:

(4) $$\begin{array}{c} Cv_{i}=\lambda_{i}v_{i}, \end{array}$$

where eigenvalues *λ*_1_ ≤ *λ*_2_ ≤ ⋯≤*λ*_*M*_ are in increasing order and **v**_*i*_, *i* = 1,…, *M* are the corresponding eigenvectors. 

In order to reduce the “bias” caused by the algorithm and length of data, amplitude adjusted Fourier transformed (AAFT) surrogate method [16] was used in this study. Based on the surrogate series *X*_surr_, the normalized phase surrogate correlation matrix **R** was calculated, and the *λ*_1_^*s*^ ≤ *λ*_2_^*s*^ ≤ ⋯≤*λ*_*M*_^*s*^ were the eigenvalues of surrogate correlation matrix **R**. The distribution of the surrogate eigenvalues can reflect the random synchronization of the multivariate time series. To reduce the effects of the random components in the total synchronization, the eigenvalues were divided by the averaged surrogate eigenvalues. The GSI was calculated by

(5) $$\begin{array}{c} \lambda_{i}^{g}=\frac{\lambda_{i}/\bar{\lambda_{i}^{s}}}{\sum_{i=1}^{M}\lambda_{i}/\bar{\lambda_{i}^{s}}}\;\left(i=1,\ldots ,M\right),\\ \text{GSI}=1+\frac{\sum_{i=1}^{M}\lambda_{i}^{g}\log\left(\lambda_{i}^{g}\right)}{\log\left(M\right)}, \end{array}$$

where $\bar{\lambda_{i}^{s}}$ is the averaged eigenvalues of the surrogate series.

Calculating the GSI used 10 s epochs with 50% overlap for the alpha rhythm over the five regions and the whole brain. Outlier's rejection [14] was also used, and then the remained epochs were averaged. Average of GSI over different regions and the whole brain was obtained as well.

#### 2.4.4. Calculation of the Relative Variable Ratio

In order to estimate the changes during the Pilates training, the relative variable ratio may be calculated by

(6) rji(k)=yji(k)−yj1(k)yj1(k)(i=1,…,N,N=6;j=1,…K,K=5;k=1,2,3),

where *N* is the number of tests, *K* is the number of subjects, and *r*_*ji*_^(*k*)^ is the relative variable ratio to the first test. *y*_*ji*_^(*k*)^ is the feature value of EEG recordings. When *k* = 1, *r*_*ji*_^(*k*)^ presents the changes of the peak power; when *k* = 2, *r*_*ji*_^(*k*)^ presents the changes of the peak frequency; when *k* = 3, *r*_*ji*_^(*k*)^ presents the changes of GSI. All changes were over the Pilates training.

If the variables increased over the Pilates training, *r*_*ji*_^(*k*)^ will be greater than zero; if they decreased, *r*_*ji*_^(*k*)^ will be less than zero; if there are no changes, *r*_*ji*_^(*k*)^ will be approximate to zero. For the limited numbers of only five subjects, boxplot is used to describe the changes over the Pilates training duration.

## 3. Results

### 3.1. Spectral Analysis

**Figure 2:**
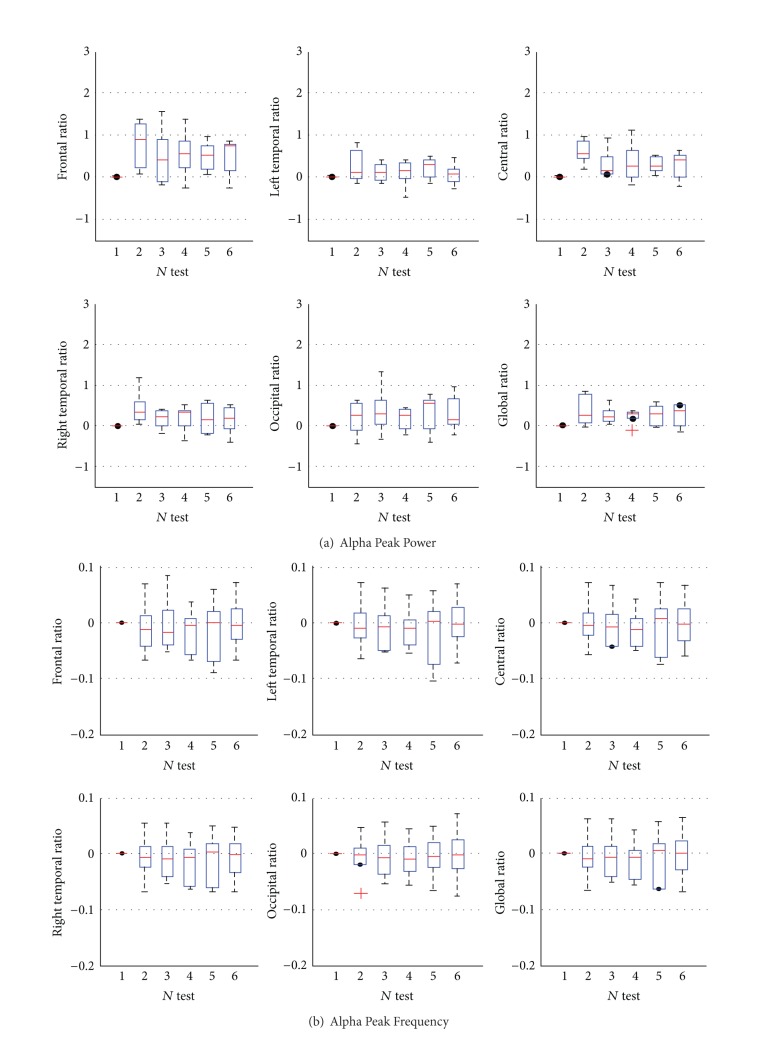
Relative changes of alpha peak power (a) and peak frequency (b) during the Pilates training. Alpha peak power increased in the five regions and the whole brain as (a) shows. As (b) shows, most of the median of alpha peak frequency decreased but was not significant. One box represented one test in (a) and (b).

The results of alpha peak power and alpha peak frequency in each region and over the whole brain were shown in Figure 2. The comparisons of global changes before training (BT) and after training (AT) for each case were shown in Table 1.

The alpha peak powers were different among the five cases. The power that is in the first case was the largest. A relative lower peak power was observed in the second and the third cases. There may be individual difference, but the trend of changes was the same. Table 1 presented that the alpha peak power increased in all cases and the average value increased as well (61.74 to 70.07 ± 10.96) (Table 1). The changes of alpha peak frequencies varied among different individuals: decreased in three cases, increased in one case, and unchanged in one case, and the average value was slightly decreased (10.29 to 10.18 ± 0.11) (Table 1).

The ratios of alpha peak power and alpha peak frequency could eliminate the effect of individual factor (see Figure 2). The ratios were obtained to investigate the two indicators' changes during Pilates training.  Figure 2(a) showed that alpha peak power was increased in various regions and the whole brain. The median of ratios was greater than zero. The ratios of alpha peak power versus alpha peak frequency were increased by about 30% to 90%, (especially in the second test, which was two weeks after Pilates training), 10% to 30%, 10% to 60%, and 20% to 40%, for the frontal, temporal, central, occipital, and the whole brain, respectively. The alpha peak frequency decreased in small degree during Pilates training, and the changes were not statistically significant (see Figure 2(b)). 

### 3.2. GSI

The GSI changes of the whole brain before and after pilates training in individuals and the average value of the five subjects were listed in Table 1. The GSI values were decreased during the Pilates training significantly.

**Figure 3:**
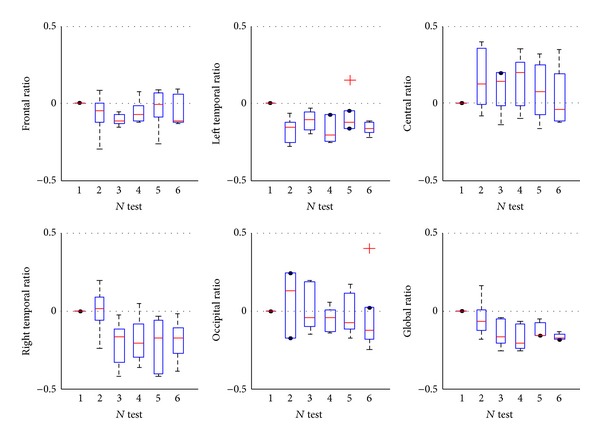
Relative changes of GSI for alpha rhythm during the Pilates training. The GSI in the frontal and temporal regions was decreased, but it almost increased in the central region, and the changes in the occipital region were not obvious. The GSI over the whole brain decreased obviously. One box represented one test.

The time-dependent changes of GSI during the Pilates training in different regions and over the whole brain were also studied.  Figure 3 plotted the relative variable ratios of GSI. For the frontal region, the GSI has decreased by about 0–10%, 8%–10%, and 5% after two, four, and six weeks training, respectively, but increased in some subjects after eight weeks training. For the left temporal region, the GSI decreased at least by 5–25% after two weeks training. For the right temporal region, the GSI decreased at least by 5–40% after four weeks training, but there was inconsistent variation after the two weeks training. For the central region, the GSI increased in varying degrees after two weeks training. For the occipital region, there were no consistent changes during Pilates training. For the whole area of the brain, the GSI decreased slightly after two weeks training but decreased at least by 5% after four weeks training. 

## 4. Discussions

In this study, we used the resting-state EEG recording to investigate the effects of the Pilates training on the brain EEG. The results showed that the Pilates training could increase the power of the brain alpha rhythm and reduce the synchronization strength of alpha rhythm in the frontal and temporal regions. These findings may support that the Pilates training maybe beneficial for improving brain function because the alpha rhythm and its synchronization are associated with the human brain higher function such as intelligence. These results suggest that Pilates training may be helpful for the intervention of brain degenerative diseases and cogitative dysfunction rehabilitation. Future study will demonstrate this hypothesis. 

Human EEG activity reflects the synchronization of cortical pyramidal neurons. Alpha rhythm in the spontaneous EEG signals is an important predictor of the efficacy of cortical information processing during cognitive and sensorimotor demand [17]. Alpha rhythm is often considered as one of the indicators of the brain function and has a significant correlation with performance on memory tasks [18], and the alpha power is considered as an important parameter to represent neural activities and processing mechanisms [19]. Although the exact mechanisms of alpha rhythm generation and its functional significance are not understood completely so far, there is increasing evidence that synchronized oscillatory activity in the cerebral cortex is essential for spatiotemporal coordination and integration of activity of anatomically distributed but functionally related neural elements [20]. Alpha power was positively correlated with intelligence variables, while some lower frequency bands negatively correlated with them [21]. The higher the absolute amplitude or power of the EEG, the stronger the background neural synchronization, then the better the cognitive performance [22], and the higher the IQ [23]. Lower alpha power is associated with many diseases, such as obsessive-compulsive disorder [24], Down's syndrome [25], Alzheimer' [26], and restless legs syndrome [27]. Patients with these diseases showed intelligence, memory loss, and alpha rhythm abnormalities [26]. There is also a correlation between alpha power and intelligence [21]. Cortical neural synchronization at the basis of eye-closed resting-state EEG rhythms was enhanced in elite karate athletes [28]. In this study, the alpha peak power was increased during the Pilates training, which suggests the increased neural network activity and perhaps the intelligence during the Pilates training. 

Previous study found that right postcentral gyrus and bilateral supramarginal gyrus were sensitive to the motor skill training [29], and the functional connectivity in the right postcentral gyrus and right supramarginal gyrus strengthened from week 0 to week 2 and decreased from week 2 to week 4. The findings in these case studies are very similar to the above results, and the functional connectivity changes based on the resting-state EEG recordings are associated with motor skill learning. Another similar study also demonstrates that the frontoparietal network connectivity increased one week after two brief motor training sessions in a dynamic balancing task [30], and there is an association between structural grey matter alterations and functional connectivity changes in prefrontal and supplementary motor areas. The GSI is a synchronization method of reflecting the multichannel synchronization strength. As shown in Figure 3, the GSI values of the alpha rhythm decreased in varying degrees over the frontal and temporal regions, increased over the central region, and decreased over the whole brain for all cases after two weeks training. The frontal and temporal regions are associated with cognition (i.e., attention and planning), and the central region is motor related. Because the Pilates can improve the balance, control, and muscle strength [7], the GSI of alpha rhythm in the frontal and temporal regions decreased when the subjects were in the resting state, in which the subjects were in a very relaxed condition, without attention and planning procession. The reduction of the synchronization strength in those regions can support what is mentioned above. This study demonstrates that the Pilates training may improve the function of control. 

## Figures and Tables

**Table 1:** Comparisons of global changes before training (BT) and after training (AT) for each case.

Persons	Changes					
	Alpha peak power		Alpha peak frequency		GSI	
	BT (*μ*V^2^/Hz)	AT (*μ*V^2^/Hz)	BT (Hz)	AT (Hz)	BT	AT
First	209.26	213.47 ± 32.79	10.05	10.02 ± 0.06	0.53	0.43 ± 0.03
Second	6.53	9.67 ± 1.27	9.23	9.76 ± 0.09	0.37	0.31 ± 0.03
Third	3.55	3.91 ± 0.52	11.89	11.48 ± 0.25	0.32	0.28 ± 0.02
Forth	45.06	65.95 ± 10.97	10.23	9.61 ± 0.08	0.35	0.32 ± 0.05
Fifth	44.28	57.34 ± 9.25	10.06	10.06 ± 0.06	0.34	0.29 ± 0.02

Average	61.74	70.07 ± 10.96	10.29	10.18 ± 0.11	0.38	0.33 ± 0.03
